# Critical examination of current response shift methods and proposal for advancing new methods

**DOI:** 10.1007/s11136-020-02755-4

**Published:** 2021-02-17

**Authors:** Véronique Sébille, Lisa M. Lix, Olawale F. Ayilara, Tolulope T. Sajobi, A. Cecile J. W. Janssens, Richard Sawatzky, Mirjam A. G. Sprangers, Mathilde G. E. Verdam, the Response Shift – in Sync Working Group

**Affiliations:** 1 UMR INSERM 1246, SPHERE “methodS in Patient-Centered Outcomes and HEalth ResEarch”, University of Nantes, University of Tours, Nantes, France; 2 Department of Community Health Sciences, University of Manitoba, Winnipeg, Canada; 3 Department of Community Health Sciences and O’Brien Institute for Public Health, University of Calgary, Calgary, AB Canada; 4 Department of Epidemiology, Rollins School of Public Health, Emory University, Atlanta, USA; 5 School of Nursing, Trinity Western University, Langley, BC Canada; 6 Department of Medical Psychology, Amsterdam University Medical Centers, Location AMC, Amsterdam Public Health Research Institute, Amsterdam, The Netherlands; 7 Department of Methodology and Statistics, Institute of Psychology, Leiden University, Leiden, The Netherlands

**Keywords:** Patient-reported outcomes, Response shift, Operationalization, Methods

## Abstract

**Purpose:**

This work is part of an international, interdisciplinary initiative to synthesize research on response shift in results of patient-reported outcome measures. The objective is to critically examine current response shift methods. We additionally propose advancing new methods that address the limitations of extant methods.

**Methods:**

Based on literature reviews, this critical examination comprises design-based, qualitative, individualized, and preference-based methods, latent variable models, and other statistical methods. We critically appraised their definition, operationalization, the type of response shift they can detect, whether they can adjust for and explain response shift, their assumptions, and alternative explanations. Overall limitations requiring new methods were identified.

**Results:**

We examined 11 methods that aim to operationalize response shift, by assessing change in the meaning of one’s self-evaluation. Six of these methods distinguish between change in observed measurements (observed change) and change in the construct that was intended to be measured (target change). The methods use either (sub)group-based or individual-level analysis, or a combination. All methods have underlying assumptions to be met and alternative explanations for the inferred response shift effects. We highlighted the need to address the interpretation of the results as response shift and proposed advancing new methods handling individual variation in change over time and multiple time points.

**Conclusion:**

No single response shift method is optimal; each method has strengths and limitations. Additionally, extra steps need to be taken to correctly interpret the results. Advancing new methods and conducting computer simulation studies that compare methods are recommended to move response shift research forward.

**Supplementary Information:**

The online version of this article (10.1007/s11136-020-02755-4) contains supplementary material, which is available to authorized users.

## Introduction

Patient-reported outcome measures (PROMs) are incorporated into clinical practice and research to assess the impact of disease and treatment from the patient’s viewpoint. PROMs may assess a range of outcomes, including for example quality of life, satisfaction with care, treatment preferences, and illness perceptions. Assessing change in PROM scores is crucial for monitoring patients’ health status and to evaluate treatment effectiveness. However, PROM change scores may be invalidated by the occurrence of response shift. Response shift is particularly relevant for self-evaluation PROMs; these are PROMs that “yield measures rating experience as positive or negative compared with an internal standard” [1].

A range of response shift definitions have been proposed. In the context of organization research, Golembiewski et al. [2] proposed a typology of change including beta change (recalibration) and gamma change (reconceptualization). In parallel, the term ‘response shift’ was proposed by Howard et al. [3] in education research to explain the discrepancy between quantitative self-reports and interviews. They defined response shift as a change in internal standards or recalibration. Sprangers and Schwartz [4] subsequently introduced a working definition that combined and expanded the definitions proposed by Golembiewski et al. [2] and Howard et al. [3]. Specifically, they defined response shift as a change in the meaning of one’s self-evaluation of a target construct that results from a change in one’s internal standards (recalibration), a change in one’s values (reprioritization), or one’s redefinition of the target construct (reconceptualization). Subsequently, Rapkin and Schwartz [5] defined response shift as a change in appraisal that can explain an unexpected change in quality of life. Finally, Oort et al. [6, 7] highlighted that when the meaning of self-evaluation changes (the working definition of response shift [4]), the relationship between change in (observed) measurement (i.e., change in PROM scores) and change in the target construct (i.e., change in the construct that the PROM intends to measure) also changes. Hence, if response shift occurs, there is a discrepancy between observed change in PROM scores and change in the target construct. If no such discrepancy exists, then response shift cannot logically have occurred. However, it should be noted that a discrepancy between observed change and change in the target construct is a necessary but not a sufficient condition for response shift to occur. If alternative explanations can be ruled out, then the discrepancy between observed change and change in the target construct can be attributed to response shift (see also Vanier et al. [8]).

Response shift research calls for carefully designed studies [9, 10] and sound methods. A variety of response shift detection methods have been developed [11, 12] and applied in various clinical settings and populations [4, 13–18] Since some of these methods not only detect but also adjust for and explain detected response shift, we prefer to adopt the more general term of response shift methods in this paper. Some studies compared different methods to assess their convergent validity [19–23]. Methods have also been compared using simulated data [24]. All methods aim to detect response shift, but differ with respect to how they operationalize response shift (i.e., how response shift is evidenced in the results), their underlying assumptions, and alternative explanations for the observed response shift effects. These different methods call the interpretation of the results in terms of response shift into question. Indeed, one may wonder to what extent we can conclude that response shift has occurred if "response-shift effects" have been inferred by different methods using different operationalizations. One of the fundamental limitations of the methodological work to date is that the various methods have not been compared with respect to their different response shift operationalizations, corresponding interpretation of results and alternative explanations.

The current work is part of an international, interdisciplinary collaboration (see Appendix for the contributing members) to synthesize extant research on response shift [8, 25, 26].The primary objective of the current paper is to provide a critical examination of response shift methods. We compare their implied definitions, how response shift is operationalized in terms of the type of response shift they aim to detect and whether they can adjust for and explain detected response shift, their underlying assumptions, and alternative explanations of response shift results. In so doing, we additionally propose further development of new methods that address some of the limitations of existing response shift methods and are expected to advance the field.

## Overview of response shift detection methods

### Inventory of methods

Based on earlier overviews [27, 28] and previous reviews, [11, 12] of response shift methods, we identified 11 methods. These include the then-test and appraisal method, so-called design methods [3, 5, 29], semi-structured interview as an example of a qualitative method [30], the Patient Generated Index (PGI) [31], and the Schedule for the Evaluation of Individual Quality of Life (SEIQoL) [32] to illustrate individualized methods, where we will further focus on the SEIQoL, and vignette studies to exemplify preference-based methods [33].

The remaining methods rely on different statistical methods. These are identified based on the previous scoping review [11] and an expository review [12]. The most recent scoping review was based on the search of seven library databases (Biomed, CINAHL, EMBASE, Medline, ProQuest, PsycINFO, and Web of Science) for the use of the terms “response shift,” “response shift effects,” “longitudinal measurement invariance,” or “retrospective bias” and was limited to articles written in English and published or accepted for publication up to December 31, 2016. Some of the statistical methods, based on the framework of latent variables that are not directly observable but rather inferred (through latent variable models) from other variables that are observed (or directly measured), include Structural Equation Modeling (SEM) [34], Item Response Theory (IRT) [16], and Rasch Measurement Theory (RMT) [24]. Other statistical frameworks encompass Relative Importance Analysis [15], Classification and Regression Tree (CART) [14], Random Forest Regression [35], and Mixed Models and Growth Mixture Models [13] (Tables 1 and 2).

**Table 1:** Response shift methods: Description, definition, and operationalization

Method	Description			Definition					Operationalization		
Then-test method (a design method*)	The then-test is an additional measurement at posttest occasion. Respondents complete the same measure as they did at pretest and posttest, but now with the instruction to re-evaluate their level of pretest functioning Group-level analysis; can accommodate subgroup analysis			*Change in meaning of self-evaluation*: Yes Discrepancy between observed and target change**: Yes, using the operationalization of “observed change” (posttest minus pretest) and “target change” (posttest minus then-test)					*Detecting*: Recalibration: pretest-minus then-test scores *Adjusting*: posttest minus then-test scores *Explaining*: including exploratory variables (e.g., in a regression model) that can explain differences between pretest and then-test sores.		
Appraisal (a design method*)	Changes in cognitive appraisal can be operationalized by the repeated administration of the QoL Appraisal Profile (QOLAP), QOLAP version 2 or the Brief Appraisal Profile [5] Group-level analysis; can accommodate subgroup analysis			*Change in meaning of self-evaluation*: Yes Discrepancy between observed and target change**: Yes, using the operationalization of “observed change” (observed QoL change) and “target change” (expected QoL change that is explained by relevant changes in health and other standard predictors of QoL)					*Detecting*: The QOLAP version 2 and the Brief QOLAP do not make a distinction in type of response shift. The domains of the QOLAP measures include, e.g., health worries, concerns, goals, mood, and spirituality.*** Direct response shift effects: how much (statistically significant) changes in appraisal explain the discrepancy between expected and observed QoL (e.g., residuals in a regression model reflecting unexplained variance) Moderated response shift effects: significant interaction effects between appraisal change scores*catalyst *Adjusting*: Not applicable *Explaining*: No (response shift effect is indistinguishable from appraisal effect)		
Semi-structured interview (a qualitative method)	Interview questions directed at eliciting respondents’ verbalizations of possible response shift effects Individual-level analysis			*Change in meaning of self-evaluation:* Yes Discrepancy between observed and target change**: Yes, dependent on the questions, interviews may elicit reflections on observed change (pretest–posttest) and change in the target construct (reflections on that change where respondents replace earlier verbalizations by new ones claiming the latter are more true)					*Detecting*: *Recalibration*: - would you have rated the level of your HRQOL in the same way at (name reference period) if asked at that time rather than now (in retrospect)? - does the response level “a ‘good’ day” (physically/socially/ emotionally/cognitively) mean a different thing now as opposed to (name reference period)? *Reprioritization*: -are some things more or less important for you now? *Reconceptualization*: - has the meaning of HRQOL changed for you? - are different things important to you now? (Questions, in part, taken and adapted from Beeken et al. [30]) *Adjusting*: Not applicable *Explaining*: respondents may provide explanations for their answers or they may be deduced from interview excerpts		
Schedule for the Evaluation of Individual Quality of Life (SEIQoL) (an individualized method)	The SEIQoL asks respondents to nominate the five most relevant domains to their HRQoL. They then assess their current functioning for each domain using a VAS ranging from best to worst possible functioning. Patients then rank the relative importance of each domain by allocating 100 points to the five domains, using a pie chart disc (judgment analysis can also be used) The SEIQoL generates an overall index score, which is the sum of all five domain products (multiplication of each domain’s weight by its corresponding level). If the SEIQoL is administered at two points in time, response shift can be assessed Group- and individual-level analysis			*Change in meaning of self-evaluation:* Yes Discrepancy between observed and target change**: No					*Detecting*: Recalibration: No Reprioritization: difference in intra-class correlation coefficients between domain weights Reconceptualization: change in frequency and content of the nominated domains over time *Adjusting*: Not applicable *Explaining*: including exploratory variables in a regression model that can explain response shift (i.e., changes in domain weights).		
Vignettes (a preference-based method)	Patients are asked to rate one or more anchoring vignettes, describing a particular (hypothetical) health state at different points in time (e.g., from poor to excellent) Group-level analysis; can accommodate subgroup analysis			*Change in meaning of self-evaluation:* Yes partly, inferred from changes in ratings of the same vignettes Discrepancy between observed and target change**: No					*Detecting*: Reprioritization: mean change in vignette ratings Adjusting: Not applicable *Explaining*: including exploratory variables in a regression model that can explain changes in vignette ratings.		
Structural Equation Modeling (SEM) (a Latent Variable Method)	Requires a longitudinal dataset with at least 2 measurement occasions. Uses the factor-analytic framework to operationalize response shift in terms of change in specific model parameters; initially developed at domain (including multiple item)-level, can accommodate individual item-level analysis Group-level analysis; can accommodate subgroup analysis			*Change in meaning of self-evaluation:* Yes, inferred from changes in the measurement and model’s parameters Discrepancy between observed and target change**: Yes, using the operationalization of “observed change” (observed change in scores) and “target change” (change in the unobserved latent variables)					*Detecting:* longitudinal non-invariance of the following model parameters: Uniform recalibration: intercepts Reprioritization: values of factor loadings Reconceptualization: pattern of factor loadings Non-uniform recalibration: residual variances *Adjusting:* change can be investigated incorporating response shift effects *Explaining*: exogenous variables can be included as covariates in SEM to explain response shifts		
Item Response Theory (IRT)/ Rasch Measurement Theory (RMT) (a Latent Variable Method)	Requires a longitudinal dataset with at least 2 measurement occasions. Response shift is indicated by change in discrimination power (one parameter per item) and difficulty parameter (p-1 parameters for an item with p response categories) Group-level analysis; can accommodate subgroup analysis			*Change in meaning of self-evaluation:* Yes, inferred from changes in the measurement and model’s parameters Discrepancy between observed and target change**: Yes, using the operationalization of “observed change” (observed change in item’s responses) and “target change” (change in the unobserved latent variables)					*Detecting*: longitudinal non-invariance of the following item parameters: Recalibration: items’ difficulties Reprioritization: discrimination power *Adjusting*: change can be investigated incorporating response shift effects *Explaining*: exogenous variables can be included in IRT/RMT models to explain response shifts		
Relative Importance Analysis	Requires a longitudinal dataset with maximally 2 measurement occasions and the a priori identification of two independent groups Two test procedures were proposed: (1) changes in discriminant analysis/logistic regression coefficients over time, and (2) changes in the rank ordering of the domains over time Uses the logistic regression or discriminant analysis framework to operationalize response shift in terms of change in the relative importance of component domains over time, in one group relative to a reference group Group-level analysis; can accommodate subgroup analysis			*Change in meaning of self-evaluation:* Yes, inferred from changes in relative importance of a domain Discrepancy between observed and target change**: No					*Detecting*: Reprioritization: statistically significant change in relative importance of a domain between two time points. *Adjusting*: Not applicable *Explaining*: exogenous variables can be included as covariates at each time point.		
Classification and Regression Tree (CART)	Requires a longitudinal dataset with at least 2 measurement occasions and baseline and clinical time-varying explanatory variables to recursively partition the data into homogeneous subgroups (nodes) with respect to the change in the PROM scores. Uses the CART framework to operationalize response shift in terms of discrepancy between clinical status and change in outcome or change in the relative importance of component domains Group-level analysis; can accommodate subgroup analysis			*Change in meaning of self-evaluation:* Yes, inferred from unexpected changes in the measurement and clinical status and/or order of importance of domains Discrepancy between observed and target change**: No					*Detecting*: Recalibration: inconsistent changes in PROM scores and clinical status Reprioritization: change in the order of importance of each domain over time *Adjusting*: Not applicable *Explaining*: exogenous variables can be included as covariates to explain change in the outcome		
Random Forest Regression	Requires a longitudinal dataset with at least 2 measurement occasion and two groups. Evaluates changes in the relative contribution of HRQOL domains to the prediction of an outcome over time in each group. The relative importance of each domain is assessed using the average variable importance (AVI), which is the relative contribution of a domain to the prediction of an outcome in a CART averaged across several bootstrap samples. The change in the AVI for each component domain in predicting a global QOL scores over time for each group is examined. Response shift is indicated by crossing curves Group-level analysis; can accommodate subgroup analysis			*Change in meaning of self-evaluation:* Yes, inferred from order of importance of domains Discrepancy between observed and target change**: No					*Detection*: *Reprioritization*: interaction between change in AVI for different domains. *Adjusting*: Not applicable *Explaining*: exogenous variables can be included as covariates to explain change in the outcome.		
Mixed Models and Growth Mixture Models	Requires a longitudinal dataset with at least 3 measurement occasions. Uses mixed models (from which the residuals are obtained, e.g., observed minus predicted HRQoL scores) followed by growth mixture models (from which latent class of homogeneous centered residuals growth trajectories are identified). Response shift is indicated by change in centered residuals over time Group-level analysis; can accommodate subgroup analysis			*Change in meaning of self-evaluation:* Yes Discrepancy between observed and target change**: Yes, using the operationalization of “observed change” (observed scores) and “target change” (predicted scores)					*Detecting*: Can detect a general response shift effect. Discrepancy between observed and predicted scores (centered residuals having a pattern of fluctuation over time deviating from zero). *Reprioritization*: Effects of domain scores on global HRQoL scores that vary with time (i.e., interaction with time). *Adjusting*: Change can be investigated incorporating reprioritization (integrating an interaction term of domain scores with time in the mixed model) *Explaining*: Exogenous variables can be incorporated to explain the pattern of fluctuations of the centered residuals.		

Recalibration: change in one’s internal standards; Reprioritization: change in one’s values; Reconceptualization: change in one’s definition of the target construct. Uniform recalibration: change in all response options in the same direction and to the same extent which will affect the observed variables' mean scores; Non-uniform recalibration: "stretch or shrink" of the scale which will also affect the observed variables' variance and the covariance between them

Detecting: how the method detects response shift. Adjusting: how the method provides change scores that accommodate, or adjust for, response shift. Explaining: how the method can explain response shift

*Design methods require study design changes (e.g., extra measures) needed to detect one or more types of response shift

**Discrepancy between observed and target change: discrepancy between observed change (e.g., change in PROM scores) and target change (i.e., change in the construct that the PROM scores intend to measure). Response shift is assumed to have occurred when observed change is not fully explained by target change

*** Appraisal: The original QOLAP distinguishes among: Recalibration (changes in standard of comparison for assessing one's experience), Reprioritization (changes in strategies for sampling experience), and Reconceptualization (changes in the frame of reference). However, the amount of residual variance explained by changes in appraisal and identified as response shift has not been translated back into the three types of response shift yet

**Table 2:** Response shift methods: Assumptions and alternative explanations

Method	Assumptions	Alternative explanations
Then-test (a design method*)	*Internal standards*: Posttest and then-test share same internal standards *Recall*: Respondents accurately recall their pretest state when completing then-test *Homogeneity*: The majority of the sample shows response shift in the same domain and same direction	Differences between mean pretest and then-test scores can also be due to response biases such as effort justification, and social desirability responding Given the need for retrospection, this method is also prone to recall bias and implicit theories of change**
Appraisal (a design method*)	*Reflection and recall*: respondents can reflect on the way they have completed questionnaire items and are able to recall it *One size fits all*: Appraisal is similar for all questionnaire items as one appraisal questionnaire is applicable to all items of a questionnaire *Likeness*: Changes in appraisal scores reflect changes in appraisal of questionnaire completion over time *Homogeneity*: The majority of the sample shows change in appraisal in the same domain and same direction	The operationalization of appraisal (e.g., health worries, concerns, goals, mood, and spirituality) does not distinguish among appraisal of HRQoL, HRQoL itself, adaptation, and response shift Given the need to retrospect on the way respondents completed questionnaire items, this method is prone to response bias such as recall bias and social desirability responding
Semi-structured interview (a qualitative method)	*Reflection*: Respondents can reflect on their quality of life, functioning, and response behavior *Verbalization*: Respondents are able to verbalize reflections *Awareness*: Respondents are aware of possible response shifts (may apply to some interview questions, not all)	Recall bias and implicit theories of change** can be introduced if interview questions ask to reflect on the past Respondents may indicate change that could be interpreted as response shift but which in fact is enforced by the interview context (e.g., response biases such as demand characteristics, social desirability responding) Response shift may remain undetected when respondents are not capable of reflection or verbalization
Schedule for the Evaluation of Individual Quality of Life (SEIQoL) (an individualized method)	*Reflection*: Respondents can reflect which domains are important to them and weigh their importance *Memory*: On repeated assessments, respondents can remember earlier mentioned domains	Change in weights (reprioritization) may be an artifact of the calculation method as they need to add up to 100. A decrease in the relative importance of one cue implies increases in the relative importance of other cues Change in domain content (reconceptualization) may be caused by forgetting to nominate a domain previously mentioned (recall bias), not listing a domain that has improved, mentioning a different domain due to implicit theory of change** or mentioning a similar domain at a different level of abstraction If used at the individual level, changes in ranking or content of domains may be attributed to chance fluctuations, such as changes in mood or just measurement error
Vignettes (a preference-based method)	*Homogeneity*: The majority of the sample shows response shift in the same domain and same direction *Likeness*: Ratings of vignettes reflect respondents’ assessments of their own health states	If vignettes describe health states outside respondents’ experience and knowledge, change in ratings over time may be caused by factors that are irrelevant to the vignettes
Structural Equation Modeling (SEM) (a Latent Variable Method)	*Homogeneity*: The majority of the sample shows response shift in the same domain and same direction *Minority*: Response shift is present in a minority of the items/variables	Misspecification of the measurement model (e.g., ignoring multidimensionality) Inter-relations between the different forms of response shift: reprioritization may in fact reflect non-uniform recalibration and vice versa Change in residual variances (non-uniform recalibration) can also be due to change in intercepts (uniform recalibration) or in factor loadings (reprioritization) going in different directions
Item Response Theory (IRT)/ Rasch Measurement Theory (RMT) (a Latent Variable Method)	*Homogeneity*: The majority of the sample shows response shift in the same domain and same direction *Minority*: Response shift is present in a minority of the items	Misspecification of the measurement model (e.g., ignoring multidimensionality) Inter-relations between the different forms of response shift: Reprioritization may in fact reflect non-uniform recalibration and vice versa Differential change in difficulty parameters (non-uniform recalibration) can also be due to uniform recalibration (or reprioritization for IRT) response shifts going in different directions
Relative Importance Analysis	*Homogeneity*: The majority of the of the sample shows response shift in the same domain and same direction within each subgroup	Relative importance of component domains is sensitive to non-normal data distributions and multi-collinearity when the analysis is conducted using discriminant analysis and logistic regression, respectively, leading to false rank ordering of the domains and false detection of reprioritization response shift Change in relative importance weights or ranks may be due to the existence of more than two observed subgroups (i.e., heterogeneity due to presence of latent groups)
Classification and Regression Trees (CART)	The clinical criterion is measured without any measurement error *Homogeneity*: The majority of the sample shows response shift in the same domain and same direction within each subgroup (i.e., data are partitioned into homogeneous subgroups (nodes))	This method might be prone to model overfitting leading to false detection of response shift
Random Forest Regression	*Homogeneity*: The majority of the of the sample shows response shift in the same domain and same direction within each subgroup (i.e., data are partitioned into homogeneous subgroups (nodes))	Random forest models are prone to overfitting leading to false detection of response shift, when not cross-validated When the autocorrelation within each explanatory domain over time is ignored, this might affect the estimated importance of each domain (i.e., average variable importance) and possibly the detection of response shift The choice of average variable importance metric can affect the rank ordering of the component domains at each occasion
Mixed Models and Growth Mixture Models	*Homogeneity*: The majority of the of the sample shows response shift in the same domain and same direction within each latent class	Misspecification of the mixed model for predictions (e.g., misspecified predictors, interactions, covariance structure) might lead to inaccurate trajectories for the residuals from which response shift is deduced Non-monotonic trajectory patterns of residuals may be attributable to other phenomena, such as cognitive impairment

The assumptions are based on specific literature (references included in the text) and general methodological knowledge. Recalibration: change in one’s internal standards; Reprioritization: change in one’s values; Reconceptualization: change in one’s definition of the target construct. Uniform recalibration: change in all response options in the same direction and to the same extent which will affect the observed variables' means; non-uniform recalibration: "stretch or shrink" of the scale which will also affect the observed variables' variance and the covariance between them

*Design methods require study design changes (e.g., extra measures) needed to detect one or more types of response shift

**Implicit theories of change: the current state of attribute or belief is assessed and a theory of stability or change is invoked

Other response shift methods have been proposed, including adaptations of the Repertory Grid Technique, Extended Q-TWiST method, Ideal Scale method, and idiographic assessment of personal goals [27]. However, these methods, requiring specific measures, have rarely been used and tested, and are therefore not included in this critical examination.

### Description

All methods have requirements regarding study design. They all demand a longitudinal design with at least two or three measurement occasions, with the exception of qualitative methods that may ask the respondent to compare the present in relation to a past reference period. The then-test is the only method needing an additional measurement in addition to the baseline and follow-up PROM; other methods require the use of specific instruments (i.e., appraisal, SEIQoL, vignettes). Some statistical methods require a priori identification of subgroups (Relative Importance Analysis) or clinical explanatory variables (CART and Random Forest Regression) (Table 1 and Supplementary Table 1).

Response shift can be investigated at the individual or at the group level (i.e., by aggregating scores). Some methods can accommodate both group- and individual-level analyses (individualized methods such as the SEIQoL that allow aggregation of individual-level data), while others are used for individual-level analysis (i.e., qualitative methods) or group- or subgroup-level analysis (e.g., then-test, Vignettes, SEM) (Table 1).

### Definitions

We chose the response shift working definition provided by Sprangers and Schwartz [4] and the definition proposed by Oort et al. [6, 7] to describe the methods. The former encompasses the definitions proposed by Golembiewski et al. [2] and Howard et al. [3], while Oort et al. adopted a different perspective, which is based on the discrepancy between observed change and target change. These definitions can be applied to all methods, whereas the Rapkin and Schwartz [5] definition requires assessing appraisal processes.

All methods operationalize the working definition of response shift, albeit indirectly, i.e., by inferring it from data. Six methods distinguish between observed change and target change, i.e., then-test, appraisal, qualitative methods, SEM, IRT/RMT, and Mixed Models and Growth Mixture Models. However, the discrepancy between observed change and target change is operationalized differently. To exemplify, qualitative methods use the discrepancy between reported change over time and modifications of that change when asked for a reflection SEM and IRT/RMT use the discrepancy between observed change scores and change in unobserved latent variables. The other methods do not distinguish between observed change and target change (Table 1).

### Operationalization

Clearly, all methods aim to detect response shift, but some methods can only detect one type of response shift. The then-test and RMT aim to assess recalibration, whereas vignettes, and Relative Importance Analysis and Mixed Models and Growth Mixture Models can assess reprioritization. IRT, CART, and Random Forest Regression aim to assess both recalibration and reprioritization, the SEIQoL is targeted at assessing reprioritization and reconceptualization, and qualitative methods and SEM aim to assess all three types of response shift. Only the appraisal method does not distinguish among type of response shift and assesses an overall response shift effect (Table 1).

Some methods can adjust for response shift, i.e., assess change while controlling for response shift. These include the then-test, SEM, IRT/RMT, Mixed Models and Growth Mixture Models. All methods, with the exception of appraisal, can accommodate exogenous variables (e.g., sociodemographic, clinical, and psychosocial variables) aimed to explain potential causes of the detected response shift effects, by for example including these variables as covariates in statistical models (Table 1).

#### Assumptions

Some methods require reflection from the respondents on their Health-Related Quality of Life (HRQoL), functioning, and/or response behavior (i.e., appraisal, qualitative interviews), and/or life domains that are important to them and their relative importance (i.e., individualized methods such as the SEIQoL) (Table 2). Qualitative methods additionally require that respondents are able to verbalize such reflections and, dependent on the question, may be assumed to be aware of possible response shifts. The preference-based methods based on vignettes assume that ratings of vignettes can be translated into respondents’ assessments of their own health state. The ability to recall is an important assumption for the use and interpretation of a number of methods, including the then-test (previous level of functioning), appraisal (previous completion of questionnaire items), qualitative methods (a past reference period), and individualized methods such as the SEIQoL (previous domains or weights).

The methods that can only detect response shift at group or subgroup level (i.e., then-test, appraisal, vignettes, SEM, IRT/RMT, Relative importance analysis, CART, Random Forest Regression, and Mixed Models and Growth Mixture Models) assume that response shift is more or less homogeneous within a (sub)sample and affects the majority of the respondents in the same domain (or items) and in the same direction. SEM and IRT/RMT additionally assume that response shift is only present in a minority of the items/variables (Table 2).

### Alternative explanations

All methods are prone to the possibility of alternative explanations of the response shift results, albeit different ones. Many influences can lead to the conclusion that response shift is present when it in fact is not, or vice versa, including recall bias (i.e., then-test, appraisal, qualitative interviews, and individualized methods such as the SEIQoL), response bias such as social desirability responding (i.e., then-test, appraisal, qualitative methods), the incapacity of verbalizing one’s own experience and feelings (i.e., qualitative methods), and more generally irrelevant “stimuli” for the patients (e.g., interview questions, and health state descriptions in the vignettes).

For all statistical methods, misspecification of the (measurement) model can lead to the conclusion that response shift has occurred when it has not, or vice versa. For SEM and IRT/RMT methods, inter-relations between different types of response shift may also lead to falsely concluding that one type of response shift has occurred (e.g., reprioritization) while in fact another type has (e.g., recalibration) (Table 2).

### Future research

This review reveals limitations of the current response shift methods. We address the need to cautiously interpret the results of the response shift methods. Since response shift is not likely to occur in the entire group under study with the same type, magnitude, and/or direction, we suggest advancing new methods for handling inter-individual variation in change over time. Moreover, the course of response shift is often unknown and may not exclusively occur within two measurement occasions. We suggest new methods that can accommodate multiple time points.

### Interpreting results as response shift

When response shift can be inferred by one of the methods (Tables 1 and 2), extra steps need to be taken to exclude alternative explanations or make these less plausible, to correctly interpret the results as response shift as defined by Sprangers and Schwartz [4]. For example, for the methods testing the discrepancy between observed and target change [6, 7], there is particular confusion about how to interpret detected response shift effects. This operationalization may indeed be a necessary but not sufficient condition for response shift to occur allowing alternative explanations for this discrepancy. For example, imagine that the item relating to the assessment of the most acute pain is one of the indicators that measure pain severity. Suppose that patients rated their acute pain to be lower at follow-up, even though their general pain severity did not change or changed to a lesser degree. This result indicates a discrepancy between observed change and target change. A possible explanation for this result may be that patients adapted to the experience of pain. It may also be that patients received pain killers that only reduced acute pain. One could argue that only the first interpretation coincides with the working definition of response shift as a change in the meaning of one’s self-evaluation of a target construct as a result of recalibration [4]. Hence, examination of possible alternative explanations of the findings need to be considered and ruled out before the conclusion that response shift has occurred is warranted.

### Inter-individual variation

New insights about adaptations to health events could be gained through exploration of heterogeneity in response shift, where different people experience different types and/or directions of response shift, or no response shift. Such insights could for example be used to develop new treatments and interventions for patients. However, most response shift methods typically assume response shift to be homogeneous, ignoring that interpretation of items may be influenced by cultural, developmental, or personality differences, contextual factors or life circumstances, and/or because of different health experiences or events.

A number of methods have been forwarded to aid the identification of variation in response shift between individuals (Table 3). For example, inter-individual variation, based on residuals, was examined using Mixed Models and Growth Mixture Models [13]. Among some challenges encountered with this method, alternative explanations, other than response shift, for the discrepancies in the direction of the residuals were cited by the authors. Other studies have explicitly investigated the effect of measured covariates on response shift in longitudinal data. For example, King-Kallimanis et al. [36] demonstrated the use of SEM with age, sex, and CD4-cell counts as covariates, to explain variation in response shift on physical and mental HRQoL of HIV/AIDS patients. The authors however noted some challenges which included fitting multiple models to the data and sample size requirements. Lix et al. [37] approached inter-individual variation by stratifying their SEM analysis, according to disease activity, an a priori known source of heterogeneity. The authors found evidence of different types of response shift in these different groups and noted that this approach is useful when only a small number of measured covariates are investigated. Salmon et al. [22] proposed using a combination of Mixed Models and Growth Mixture Models and SEM to detect response shift among a cohort of breast cancer patients who provided self-reported cancer-related fatigue. Latent classes that showed different types of response shift were identified. The authors however noted some concerns, including mixed model misspecifications [38] and ignoring the uncertainty of classification for the latent classes [39, 40].

**Table 3:** Response shift methods for inter-individual variation

Method	Description	Challenges
Mixed Models and Growth Mixture Models	Using mixed models (from which the residuals are obtained) followed by growth mixture models (from which latent class of homogeneous centered residuals growth trajectories are identified). Response shift is indicated by change in centered residuals showing a pattern of fluctuation over time	Mixed model misspecifications can bias predictions and, hence, residuals. Potential contributing factors other than response shift might influence the discrepancies in the direction of the centered residuals, e.g., cognitive impairment [13]
Structural Equation Model (SEM) with covariates	Testing covariate effects directly within SEM to investigate their effects on response shift in longitudinal data	Fitting multiple models to the data requires a sufficiently large sample size to provide adequate statistical power to detect covariate effects and ensure sufficient heterogeneity to identify subgroups that may experience different types of response shift [36]
Structural Equation Model with stratification	Stratified SEM analysis, according to an a priori known source of heterogeneity, e.g., disease activity in inflammatory bowel disease	Only a small number of measured covariates can be investigated at the same time [37]
Mixed Models and Growth Mixture Models and Structural Equation Model	Combination of Mixed Models and Growth Mixture Models and SEM to detect response shift and address potential heterogeneity in the types of response shift. Can be applied when the sources of heterogeneity are unknown a priori. They can then be inferred from data using a latent class approach	This approach results in multi-step analyses with a cascade of statistical manipulations that can raise concerns, including mixed model misspecifications (e.g., predictions can be affected by misspecified covariance structure) and ignoring the uncertainty of classification for the latent classes in SEM where the feasibility and performance of recommendations coming from mixture modeling are unknown in SEM for response shift analyses [22, 39, 40]
Latent Variable Mixture Models (LVMMs)	LVMMs examine heterogeneity when there is no prior information on measured covariates that may contribute to patient differences. Heterogeneous samples are stratified into groups that are similar by specifying latent classes in the measurement model	These models need to be extended to longitudinal data by examining the possibility of latent classes with different over-time constraints on measurement model parameters that represent different types of response shift (or no response shift). The computational resources required to estimate a large number of model parameters may need to be secured [12]

Clearly, new tools are needed to explore inter-individual variation in response shift especially when the sources of variation are not known a priori. Latent Variable Mixture Models (LVMMs) have been put forward for this purpose [41]. Sawatzky et al. [42] proposed the use of LVMMs models to identify homogenous samples with respect to a unidimensional measurement model, and described the implications and sources of sample heterogeneity. However, challenges to extend these models for response shift analysis remain [12] and further research is needed to examine how well LVMMs perform in longitudinal data and to test implications of various specifications (e.g., whether to correlate item residual variances over time) and secure computational resources [43, 44].

### Multiple time points

Response shift analysis is particularly relevant after the occurrence of a significant health event (catalyst; [4]). Hence, many studies have employed two measurement occasions: before and after such an event (e.g., a diagnosis, a treatment). Whereas such an approach may be justified if one wishes to study the effect of a clearly identified health event, limiting response shift detection to only two measurement occasions may be too restrictive in other instances. For example, some health state changes may have gradual shifts either or not in combination with sudden shifts, such as in recovery processes. Response shift could also be the result of the mere passage of time [4]. In these instances, the use of multiple time points may facilitate the detection of response shift effects.

Response shift detection methods that can accommodate multiple time points are scarce but some have been suggested (Table 4). Oort’s [34] longitudinal SEM approach was extended to four time points and a clinical application in stroke patients was provided [45]. Some challenges related to sample size were however noted.

**Table 4:** Response shift methods for multiple time points

Method	Description	Challenges
SEM	Extension of Oort’s [34] longitudinal SEM approach to more time points, in this case four, to give insights into the timing of response shift. Inclusion of exploratory versus theory-driven model assessments, model validation, and correction for multiple testing have also been proposed as well as suggestion of a framework to assist researchers in evaluating response over multiple occasions	Large sample sizes are needed to accommodate many parameters and to avoid model overfitting [45]
Longitudinal three-mode SEM model with Kronecker product restrictions	Extension of Oort’s [34] longitudinal SEM approach to construct parsimonious SEM models for multivariate longitudinal data with many measurement occasions. Use of curves to facilitate interpretation of change in response shift parameters in the three-mode model, which includes the subjects, the variables, and the measurement occasions. This method can operationalize the different types of response shift and assess change in common factor means after accounting for potential response shift	Model parameters are assumed to change proportionally over time; accordingly, the model is best suited to data with a fixed interval between measurement occasions. Evaluation of model fit is complicated by multiplicative constraints [46, 47]
Mixed models	Mixed models can accommodate multiple measurement occasions to explore reprioritization response shift by evaluating changes in the importance of components domains to overall, e.g., HRQoL, over time (i.e., significant interaction effects with time)	This approach can be impacted by strong correlations among predictor variables. Such multi-collinearity can be checked and accounted for to avoid unreliable and unstable estimates of regression coefficients and hence spurious findings of reprioritization [48, 49]
Bayesian joint growth models	Bayesian joint growth models with random occasion-specific parameters for both the latent variable and item parameters to investigate time effects on the occasion-specific item parameters and on the latent variable simultaneously	Specifications of proper prior distributions for the latent variable and for the item parameters are needed, which might be difficult because we do not usually have a clear idea of their a priori distributions [51]

*SEM* Structural Equation Mode, *IRT* Item Response Theory, *RMT* Rasch Measurement Theory

Another extension of Oort’s [34] SEM approach is the longitudinal three-mode SEM model with Kronecker product restrictions [46, 47] which can accommodate many measurement occasions. Some challenges have nevertheless been put forward by the authors including the evaluation of model fit.

Mixed models can also accommodate multiple measurement occasions to explore reprioritization response shift [48]. This approach can however be impacted by strong correlations among predictor variables but there are solutions to overcome such multi-collinearity which can be implemented [49].

Methods particularly suited for item-level analysis, such as IRT and RMT [16, 24, 50], can also be extended to accommodate multiple time points. However, this may lead to models with numerous parameters to estimate and to model specification challenges in modeling item dependencies over time [24, 50]. Alternative modeling strategies are needed.

Bayesian joint growth models [51] may be used, which require specification of proper prior distributions. Currently, such an approach or [52] a frequentist multiple time points framework not requiring prior knowledge is being explored. Irrespective of the chosen framework, interpreting and translating the trajectories of the item parameters to the working response shift definition might be a challenge. Moreover, methods for identifying key time points where we could consider that a clinically meaningful change in items’ parameters has occurred might also be needed. Joint models with change of the response shift parameters [53] that allow handling interval censoring could be promising since the occurrence of response shift is assumed to lie within an interval without being observed exactly [54].

## Epilogue

Despite operational differences among the eleven response shift methods, they share a common working definition related to a change in the meaning of one’s self-evaluation of a target construct [4]. Not surprisingly, this critical examination showed that no single response shift method is optimal in all situations; each has its strengths and limitations. Therefore, it is too early in the development of the field of response shift research to provide a priori recommendations regarding which method to use in a certain situation. The key finding is that response shift results cannot be accepted at face value and extra steps need to be taken to correctly interpret the results. This calls for training in the application of response shift methods and carefully designed studies. First, a rationale for the occurrence of response shift is needed that is based on substantive theory and/or clinical knowledge. Second, a precise objective needs to be formulated detailing the problem that will be examined. For example, will response shift be addressed as measurement bias that needs to be adjusted or as an interesting phenomenon in itself, shedding light on change per se. Third, wherever possible, hypotheses need to be formulated a priori about which type of response shift is expected, in which direction, in which domain, within which time period(s), and for which subsample. Fourth, methods need to be selected that correspond with the study objective and the characteristics of the data that will be collected. For example, whether the aim is to investigate domain-level, item-level response shift, or both will guide the selection of the method. Fifth, alternative explanations need to be identified for which the current overview per method is expected to be helpful. However, they can also be debated and it may be difficult to disentangle response shift from alternative explanations. For example, dimensionality and heterogeneity can also explain the discrepancy between observed and target change. Sixth, studies must be designed so that it is possible to rule out alternative explanations, or make them less plausible. This includes incorporating a control or comparison group into the design and external criterion measures of change (e.g., self-reported transition scores). We also recommend adopting the response shift method that is most appropriate for a given study (e.g., statistical methods versus individualized methods). If possible, a qualitative method may be added to investigate the causes of response shift or to provide supporting evidence [27]. However, these methods may not be suited for triangulation of group-level analytical methods, given their individual-level analytical perspective. Finally, response shift is frequently examined through secondary analysis of existing data using statistical methods. Careful attention should be given to the optimal implementation of such analyses. We refer the reader to the guidelines proposed by Schwartz et al. [9].

The current critical examination also highlighted the need to further develop new methods to investigate inter-individual variation in change over time and across multiple time points. We presented a range of promising techniques. It should be noted that while these new methods may be useful and help to advance the field of response shift research, they are not likely a panacea as they also need to be scrutinized for their susceptibility to alternative explanations. An additional way forward is to apply different methods to the same dataset to see how the methods compare with respect to detecting the same type of response shift. While this seems to be a valuable approach, it has two main caveats. First, because we do not know the “truth” (i.e., whether response shift is present or absent in the population), the findings will not tell us whether the different methods are sensitive to detect response shift. Second, different methods require different data. In a head-to-head comparison of three statistical approaches using a single dataset of patients with multiple sclerosis, it was necessary to accommodate the data to meet the requirements of each statistical approach, thereby hampering direct comparisons [21]. Perhaps a more promising path forward involves the conduct of simulation studies, which can provide insight into the performance of the extant and future methods, given known response shift effects and based on requisite simulated data that enable the comparison of the methods [55] (e.g., adequate control of Type 1 error, sufficient statistical power, examining bias). Importantly, such studies would be able to examine the effects of falsification [25] by generating simulated data that are inconsistent with response shift and examining the extent to which the methods are able to rule out alternative explanations. The use of simulation studies would also allow the assessment and the comparison of the robustness of different methods to certain model assumptions (e.g., normal distribution) and/or missing data, particularly missing not at random (MNAR) data which are likely in longitudinal studies with patients [56].

It is our hope that this critical examination will stimulate research on the further advancement of response shift research by carefully applying extant and novel methods, cautiously interpreting their results, and conducting simulation studies of extant and novel methods.

## Supplementary Information

Below is the link to the Supplementary Information.

The members of the Response Shift – in Sync Working Group (DOCX 15 kb)

Table S1. Response shift methods: Example studies (DOCX 18 kb)
