# Supplementary material for: Critical examination of current response shift methods and proposal for advancing new methods
Source: Qual Life Res. 2021 Feb 17;30(12):3325–42. doi: 10.1007/s11136-020-02755-4 (PMC8602164; doi:10.1007/s11136-020-02755-4)
Supplement: Supplementary file 2 — Table S1. Response shift methods: Example studies (DOCX 18 kb) [file 11136_2020_2755_MOESM2_ESM.docx]

**Supplementary Table 1**

**Table S1. Response shift methods: Example studies**

| **Method** | **Example** | |  |
| --- | --- | --- | --- |
| Then-test method  (a design method) | Cancer patients (N=173) were administered the EORTC QLQ-C30 at hospitalization (pretest) and three months after being discharged (posttest and then-test).  Reference: Preiss et al., Eur J Cancer Care, 2019 [23]  *Recalibration:* Patients retrospectively rated their pretest functioning as worse than they did at time of pretest. The largest effect sizes for these recalibration response shift effects (> 0.50) were found for physical functioning, role functioning, fatigue and pain.  *Adjusting:* Mean posttest minus then-test scores indicated larger improvement than mean posttest minus pretest scores | |  |
| Appraisal  (a design method) | HIV+ Medicaid recipients (N=394) were administered the SF-36 and the QOL Appraisal Profile (QOLAP) at baseline and 6 months later.  Reference: Li & Rapkin, 2009 [14]. *Response shift*: Respondents were classified into increasingly homogeneous subgroups with similar changes in cognitive appraisal profiles using Classification and Regression Trees (CART) with the aim to identify prevalent patterns of cognitive changes that can account for residual variance in QOL change scores (mental component summary (MCS) score). Standardized residual scores controlling for baseline MCS, demographics, personal history (e.g. drug use), baseline health status, baseline frame of reference, sampling standards, combinatory algorithm, change in health status, number of self-reported symptoms, and intervening care events were computed. Subgroups of response shift with the largest positive or negative residuals seemed to be mostly influenced by changes in emotion (e.g., ‘‘negative experiences  and feelings’’), subjective norms (“comparing with others”) in conjunction with the salience of negative event, frames of reference,  and individual concerns. No distinction was further made in type of response shift (i.e., recalibration, reprioritization, and reconceptualization).  *Adjusting*: Not Applicable | |  |
| Semi-structured interview  (a qualitative method) | Stem cell transplant survivors (N=28) were interviewed at one point in time, using the questions listed in Table 1, last column; e.g. *Recalibration*: does the response level “a ‘good’ day” mean a different thing now as opposed to (name reference period)?; *Reprioritization*: are some things more or less important for you now?  Reference: Beeken et al., QLR, 2011 [30] *Recalibration*: Selected patients (N= 15; 54%) provided narrative examples by describing how they would not have evaluated their HRQoL in the same way prior to transplant.  *Reprioritization*: Selected patients (N=16; 57%) described what was previously important to their HRQoL and what was now less important (e.g., work, money) or had become more important (e.g., family and friends). *Reconceptualization*: Unequivocal evidence about this type of response shift was not found. *Adjusting*: Not Applicable | |  |
| Schedule for the Evaluation of Individual Quality of Life (SEIQoL)  (an individualized method) | Patients (N=117) completed the SEIQoL at baseline and 3 months after receiving high quality dentures. In this study, the SEIQoL was combined with the then-test method, such that it was administered at follow-up as a posttest and then-test where patients were given the same domains as they had nominated at baseline and were asked to reevaluate their baseline functioning regarding these domains (recalibration) and re-evaluate cue weights (reprioritization).  Reference: Ring et al., Health Qual Life Outcomes, 2005 [32]  *Recalibration*: At the then-test, patients rated the levels of the domains differently than they did at baseline.  *Reprioritization*: At the then-test patients gave significantly different weights to the most and least important domains compared to baseline.  *Reconceptualization*: 81% of patients mentioned at least one different domain at follow-up than at baseline.  *Adjusting*: Mean posttest minus then-test Index scores indicated a significant improvement whereas mean posttest minus pretest scores were insignificant. | |  |
| Vignettes (a preference-based method) | Prostate cancer patients (N=52) were administered three vignettes at pre-diagnosis, 1, and 7 months post-diagnosis. The vignettes, describing a health state relating to treatment side effects of localized prostate cancer, included urinary, bowel or erectile function, respectively. The vignettes were evaluated on a scale from 0 ‘very bad’ to 10 ‘very good’.  Reference: Korfage et al., 2007 [33]  *Reprioritization:* As expected, the vignettes were rated significantly better at post-diagnosis than at pre-diagnosis with small to moderate effect sizes. Respondents thus reprioritized these health states as they found them less detrimental when they possibly faced these side effects themselves.  *Adjusting*: Not applicable | |  |
| Structural Equation Modeling (SEM)  (a Latent Variable Method) | Breast cancer patients (N=466) completed the Multidimensional Fatigue Inventory (MFI-20) before and after treatment.  Reference: Salmon et al., 2017 [22]  *Uniform recalibration* (decrease in intercepts): patients tended to report lower mental fatigue at the second measurement occasion compared to the first, given similar fatigue.  *Non-uniform recalibration*: in reduced motivation (increase in residual variance): may indicate that not all patients have been influenced to the same extent by response shift  *Reprioritization* (decrease in factor loadings): physical fatigue became less important to overall fatigue than other fatigue domains at the second measurement occasion compared to the first.  *Reconceptualization* (change in the pattern of factor loadings): not evidenced.  *Adjusting*: the mean level of fatigue significantly increased over time. |  |  |
| Item Response Theory (IRT)/ Rasch Measurement Theory (RMT)  (a Latent Variable Method) | Chronically ill patients (N=669) completed the General Health (GH) domain of the SF-36 shortly after admission and 6 months after discharge for surgery.  Reference: Guilleux et al., 2015 [16]  *Uniform recalibration* (same increase for all difficulty parameters): patients were more inclined to report that they expected their health to get worse at the second measurement occasion compared to the first given similar general health.  *Non-uniform recalibration* (differing change in difficulty parameters): patients were more inclined to answer “Poor” than “Fair” and “Very good” than “Good” for low and high levels of general health, respectively.  *Reprioritization* (increase or decrease in discrimination power parameters): some items became more or less discriminating for the measurement of GH levels at the second measurement occasion compared to the first.  *Adjusting*: the mean level of GH was stable over time. |  |  |
| Relative Importance Analysis | Patients with inflammatory bowel disease (N=357) completed the Inflammatory Bowel Disease Questionnaire (IDBQ), and the SF-36 at baseline and 6-month follow up.  Reference: Lix, et al., 2013 [15]  *Reprioritization*: The IBDQ social functioning domain and SF-36 bodily pain and social functioning domains showed evidence of response shift over the six-month period; active and inactive IBD symptom groups differed in their evaluations of bodily pain and social interactions over time.  *Adjusting*: Not applicable |  |  |
| Classification and Regression Trees  (CART) | Patients with dental hypersensitivity (N=75) completed the Dentine Hypersensitivity Experience Questionnaire (DHEQ) at screening and 8 weeks after. The final tree was developed using 75 valid observed DHEQ change scores and included the 5 subscales as independent variables, ending in 9 terminal nodes  Reference: Machuca et al 2017 (doi: 10.1186/s12874-017-0396-3)  *Recalibration*: was noted in 36.5% of the patients who rated their QoL as better despite unchanged clinical status. Recalibration in the opposite direction was noted in 14.7% of the patients who rated their QoL as worse despite having their clinical status resolved.  *Reprioritization*: Changes in the order of importance of the component domains was noted in all the five domains (coping, social, emotional, restriction, and identity domain).  *Adjusting*: Not applicable |  |  |
| Random Forest Regression | Individuals with multiple sclerosis (N=524) completed the Multiple Sclerosis International Quality of Life (MusiQOL) measure and SF-36 at baseline and every six months over a 24-month period. The severity of MS was measured using the Expanded Disability Status Scale. The severity of MS worsened in 417 (79.6%) patients and did not worsen in 107 (20.4%) patients. The random forest regression model was used to evaluate the presence of reprioritization response shift in SF-36 domains, accounting for patients’ demographic, employment status, and disease duration.  Reference: Boucekine et al 2013 (doi: 10.1186/1471-2288-13-20.)  *Reprioritization*: was identified on the mental component scores in the “worsened” group. That is, the mental component score became more important during the twelve months following inclusion, while the importance of mental and physical aspects was close at the initial evaluation.  *Adjusting*: Not applicable | | |
| Mixed Models and Growth Mixture Models | Stroke survivors (n=387) completed the EQ-5D VAS and rated their health (referred to as “reported health”). Measures of symptoms and time-varying measures of function (items of the EQ-5D Index, preference-based stroke index, MMSE, SF-36, stroke impact scale), baseline data and interaction with time were used for “predicted health”.  Reference: Mayo et al., 2008 [13]  *Reprioritization*: not evidenced  *General response shift*: seven latent classes (LCs) were identified: LC n°1: lack of response shift (i.e. stable centered residuals over time) for 67% of sample, LCs n°2 and 3: so-called “negative response shift” (i.e. centered residuals went from positive to negative indicating that over time, patients first tended to rate their health as “better than predicted” and later as “worse than predicted”) with either early (9%) or later drops (4%), LCs n° 4, 5, 6: so-called “positive response shift” differing in magnitude (ranging from 1% to 11%), LC n°7: so-called “unstable response shift (4%).  *Adjusting*: Not done |  |  |

Note: MMSE: Mini Mental State Examination. Recalibration: change in one’s internal standards; Reprioritization: change in one’s values; Reconceptualization: change in one’s definition of the target construct. Uniform recalibration: change in all response options in the same direction and to the same extent which will affect the observed variables' mean scores; Non-uniform recalibration: "stretch or shrink" of the scale which will also affects the observed variables' variance and the covariance between them. Adjusting: how the method provides change scores that accommodate, or adjust for, response shift.
